# A historical approach to scorpion studies with special reference to the 20^th^ and 21st centuries

**DOI:** 10.1186/1678-9199-20-8

**Published:** 2014-03-11

**Authors:** Wilson R Lourenço

**Affiliations:** 1 Département Systématique et Evolution, Muséum National d’Histoire Naturelle, UMR7205, CP 053, 57 rue Cuvier, 75005 Paris, France

**Keywords:** Scorpion studies, Historical approach, 20^th^ century, 21^st^ century, Taxonomy, Biology

## Abstract

This work provides historical context about scorpion studies from the end of the 19^th^ century to the present day. The content is mainly addressed to non-zoologists, working in research fields that embrace scorpion biology, notably to those working with venoms and toxins. The historical aspects described include academic professional scholars who worked on scorpion classification and general distribution patterns; and to a lesser extent, on studies of ecology and natural history. The aim is not to provide an exhaustive description of all scholars who in one way or another became involved with scorpions, but rather of those who greatly contributed during a given period to the research of these organisms. No critical analysis of the work of previous researchers is undertaken, but some comments are proposed to bring clarification on ‘who’s who’. Since a global consensus in relation to classification and/or distribution patterns has not been reached among modern experts, these different approaches are also presented without judgment. Consequently, distinct approaches remain open for discussion.

## Introduction

From the very beginning of this article one question may be asked: Why limit the historical description to the period from the final years of the 19^th^ century through the present? Two main reasons may justify this selected period. First, the end of the 19^th^ century marked the beginning of the decline of the ‘golden age’ of scorpion studies and equally saw the publication of the first global monograph about scorpions, *Das Tierreich* by Kraepelin [1]. Second, this period also corresponds to the emergence of interest in the venom of scorpions and was marked by the beginning of antivenom therapy [2,3]. Since this article is primarily addressed to non-zoologists, especially those working on scorpion venoms and toxins, the choice of this period seems relevant.

The monograph published by Kraepelin [1] was the first complete survey of the world’s scorpion fauna. Prior to this contribution, other authors attempted to synthesize a compendium of all the known fauna, such as Koch [4] who cited four families and 11 genera, and Peters [5] who listed four families and 19 genera. Part of the classification by Kraepelin in *Das Tierreich*[1], which included six families and 64 genera, remained almost unchanged for several decades.

The basic idea in this article is to bring some historical information about the activities of academic-professional scholars who worked on scorpion classification and biogeography and to a lesser extent the biology and ecology of scorpions from the end of the 19^th^ century to the present day. However, no attempt has been made to bring an exhaustive description of all scholars who in one way or another became involved in scorpion research. Comments are limited to those whose results greatly contributed to the research of these organisms. In the same manner, critical analysis of the work performed by previous researchers is avoided herein, but some comments are proposed to clarify ‘who’s who’ in the field of scorpion studies. Since a global consensus in relation to classification and/or distribution patterns does not exist among modern experts, these different approaches are also discussed without judgement. Consequently, distinct approaches remain open for discussion. For more general details refer to Lourenço [6].

### 1899 – 1914: the publication of Krapelin’s *Das Tierreich* and the decline of the golden age

The year 1899 was marked by the publication of Kraepelin’s *Das Tierreich*, which announced the start of a decline in what can be called the golden age for scorpion studies [7]. This period which started around the turn of the 20^th^ century, was marked by numerous contributions to the knowledge of scorpion taxonomy and biogeography.

Major contributions to the classification of scorpions have been proposed by authors such as Peters [5], Thorell [8], Simon [9,10], Kraepelin [1,11], and Pocock [12,13]. Thorell published until 1894 and died in 1901. Simon published papers in the early 20^th^ century, but from 1910 until his death in 1924, his work focused other zoological groups. Kraepelin had numerous publications during the first decade of the 20^th^ century, and died in 1915. Pocock retired from arachnology in 1903 and assumed other responsibilities as director of the Zoological Garden of London. It is assumed that his departure was due to personal problems he faced in the British Museum of Natural History. Before his death in 1947, he returned to research but only focused on other zoological groups. A number of less conspicuous contributions were also published by several authors during this period, such as those by Banks [14], Pavesi [15] and Penther [16]. Preceding the publication by Kraepelin [1], some pioneering publications, escaping from the traditional external morpho-anatomical approaches, were produced by Laurie [17,18]. These publications dealt with the reproductive anatomy of female scorpions.

### 1918 – 1938: the period between the World War I and World War II

Although the beginning of the 20^th^ century featured the retirement of several golden-age authors, it also saw the emergence of a group of scorpion researchers whose studies would continue until just before World War II. Among the most conspicuous was Bialynitskii-Birula [19,20] who published from 1896 to 1937, including some notable articles, and Borelli [21,22], with contributions from 1899 to 1934. Furthermore, additional pioneering work was conducted [18]. The most significant works are those produced by Pavlovsky [23,24] regarding the internal male genitalia. This author published mainly from 1916 to 1934.

This interwar period was also marked by a diminution in the European research hegemony that had dominated during the 19^th^ century, and by the appearance of students in several regions of the world. For example, Baerg [25], Banks [26] and Chamberlin [27] in the United States, Hoffmann [28,29] in Mexico, Hewitt [30] in South Africa, Glauert [31] in Australia, Takashima [32] in Japan, Mello-Campos [33], Mello-Leitão [34] and Lutz and Mello [35] in Brazil; these latter authors are well known for their description of the infamous Brazilian scorpion *Tityus serrulatus*.

Although the activities of European scholars had started to decline, a number of authors were still contributing to scorpion studies, such as: A. S. Hirst in the United Kingdom, L. Berland, L. Fage, H. Foley, P. Pallary and E. Sergent in France, L. di Caporiacco in Italy, L. Giltay in Belgium, J. Hadzi in the former Yugoslavia, B. P. Franganillo in Spain, E. Schenkel and A. Monard in Switzerland, C. F. Roewer in Germany and F. Werner in Austria. J. Vellard, a French arachnologist working in South America, contributed two remarkably detailed publications in 1932 and 1934 as described by Lourenço [36]. Finnegan [37], from the United Kingdom, was the first lady researcher to describe a new scorpion genus [38].

As already mentioned by Bonnet [7], the period that followed the golden age had less impact on arachnological studies than had the previous decades. The quality of the results was in many cases ‘poor’ and a rather conservative classification prevailed, so no significant changes took place in the general classification of scorpions during this time.

### 1945 – 1965: a slower pace in scorpion studies and a new emerging period

The period of scorpion research between World War I and II is often considered less significant than research conducted during the golden age. Nevertheless, it was precisely in the very last years of this epoch that some highly esteemed scorpion researchers started to emerge.

The work of Mello-Leitão in Brazil [39], continued during the war, and lasted until his death in 1948. Meanwhile, he published the impressive monograph about South American scorpions. In the United States, H. L. Stahnke started to publish in 1940 and his activities lasted until the 1980s. R. F. Lawrence from South Africa began his studies in the 1920s, and these continued until the 1960s.

**Figure 1:**
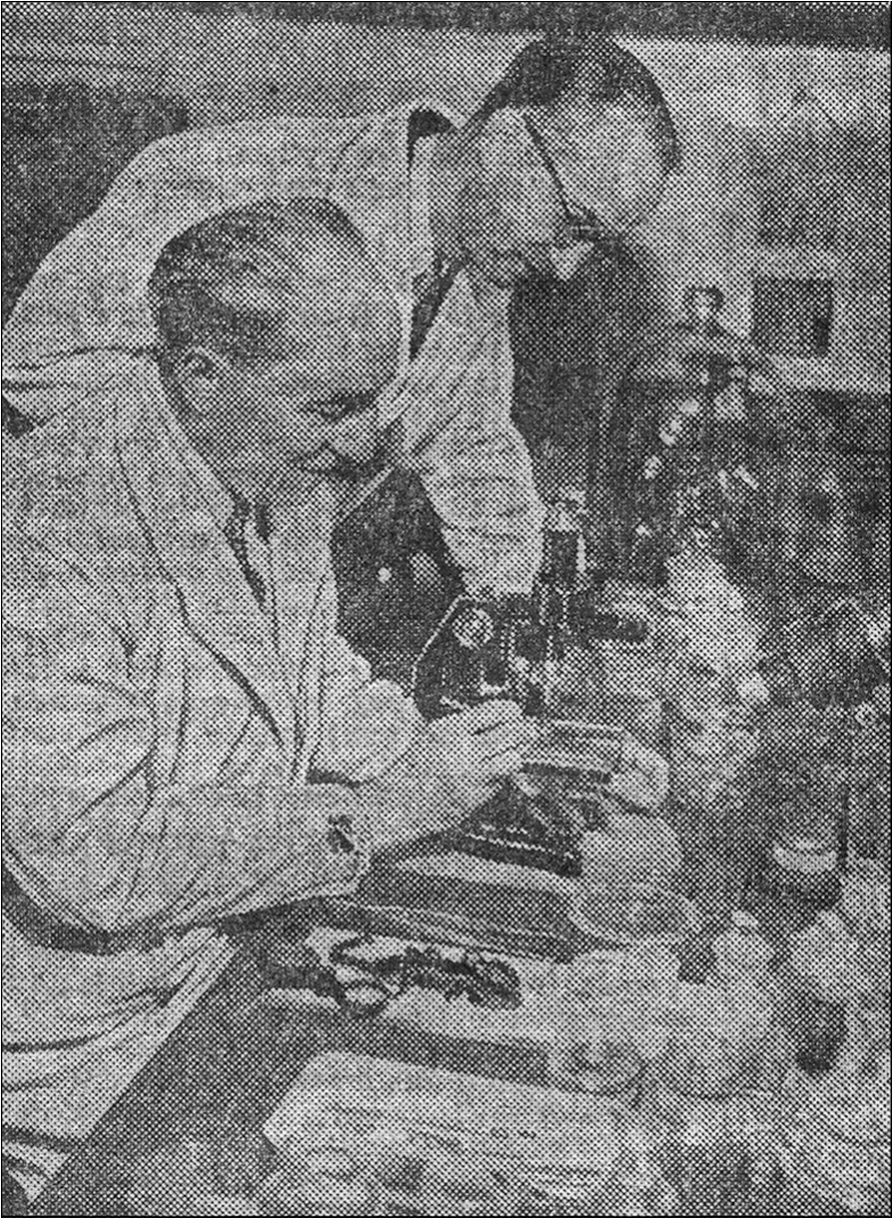
**Very old photo showing Louis Fage (seated) and Max Vachon (standing behind him), examining scorpions in the National Museum of Natural History, Paris.** Photo published in the newspaper *Le Figaro Litteraire* on July 19, 1952.

It was, however, in France that a young biologist, M. Vachon [40], was introduced to scorpion classification just before World War II. Today, it is globally accepted by most biologists that Vachon was one of the most innovative and influential scorpiologists of the 20^th^ century. After the conclusion of his biological studies in the University of Dijon, Vachon came to Paris where he obtained his Doctor’s Science degree in 1938. Surprisingly, however, the subject of his doctoral thesis was the reproductive biology of pseudoscorpions rather than scorpions or taxonomy. Shortly after getting his degree, Vachon integrated the Laboratory of Worms and Crustaceans, then directed by Professor Louis Fage, in the National Museum of Natural History in Paris. Once he became assistant professor in the Museum, L. Fage suggested that he studied scorpions (Figure 1). The main reason for this was because scorpions constituted a serious health problem in several regions of North Africa, a region that was under French administration. In North Africa there are several scorpion species that are harmful to humans, belonging to the genera *Androctonus* Ehrenberg, 1828, *Leiurus* Ehrenberg, 1828 and *Buthus* Leach, 1815. Since Simon’s death in 1924, only L. Fage continued scorpion studies, but these were rather marginal among his other activities. Consequently, he appointed M. Vachon to take care of these projects. The presence of a permanent scorpion expert in the Museum in Paris was strongly requested by Dr. E. Sergent who was facing the problems of scorpionism present in North Africa.

Vachon’s knowledge of scorpion taxonomy was extremely limited; however, he took over the task of learning about this group of organisms, and by the early 1940s he published the results of his preliminary studies [41,42]. As for the problem of scorpion taxonomy in North Africa, Vachon began a series of studies which he has published since 1948. A total of 11 articles were finally condensed into a single monographic work published in 1952 [43]. Although Vachon dedicated all his efforts to scorpion research during the 1940s and 1950s, pseudoscorpions remained his primary passion. Only by the 1960s did scorpions finally become the main focus of his research. In 1955 Vachon succeeded L. Fage as the director of the Laboratory of Worms and Crustaceans which later became known as the Laboratory of Zoology Arthropods in 1960. Vachon directed this laboratory until his retirement in 1978. After World War II (during the 1950s and 1960s), with the exception of the studies performed by Vachon, research on scorpions can be considered rather limited. Regional work was pursued by a number of scholars such as H. Stahnke and W. J. Gertch in the United States, W. Bücherl in Brazil, A. Shulov in Israel, J. V. Scorza in Venezuela and A. Diaz Nájera in Mexico. It was, however, by the end of the 1960s and in the early 1970s that a new generation of scholars appeared, some of which were trained by Vachon.

### 1966 – 1990: a new generation of professional scorpion experts takes over

From the first years of the 1960s, a young biologist from Uruguay, Pablo San Martin, started to use male internal genitalia to elucidate the taxonomy of the family Bothriuridae. He started his training, with Sylvia Lucas and Vera R. D. von Eickestedt, under the direction of Dr. Wolfgang Bücherl at the Butantan Institute in São Paulo, and subsequently pursued his studies in Uruguay. However, he died tragically and was not able to complete much of his research. His unfinished projects were taken over by E. A. Maury from Argentina who followed the same approach started by San Martin. During this period, F. Matthiesen [44], also from Brazil, carried out interesting studies on the reproductive biology of scorpions and demonstrated the existence of parthenogenesis in *Tityus serrulatus*, a phenomenon previously unknown to the group. Both of these authors later went to the Laboratory of Zoology Arthropods in Paris to be trained by Vachon.

In the United States, authors such as S. C. Williams, J. R. Reddell and R. W. Mitchell contributed a number of interesting discoveries on the North American scorpion fauna, including the description of the first true troglobitic species found in Mexico. In European countries authors were scarce during the 1960s, with some exceptions such as R. Kinzelbach in Germany and R. P. Sreenivasa-Reddy in France, who worked under Vachon’s guidance for some years, but tragically died before obtaining his Doctoral degree. In Israel, Levy [45] started important studies on the scorpion fauna of Palestine which were summarized by the significant monographic work *Fauna Palaestina. Arachnida I: Scorpiones*.

By the early 1970s a different approach was observed among scholars receiving adequate training in scorpion studies. In addition to their training they also started to prepare their doctoral dissertations using scorpions as specific tools. In the United States, Francke [46] dealt with taxonomy of the family Diplocentridae while R. Farley, Polis and Brownell [47,48] started to pave the way in scorpion ecology and ecophysiology. Shortly afterwards, Sissom [49] received his PhD and also began working on the taxonomy of North American scorpions. By the end of the 1980s, Stockwell [50], working on the phylogeny of scorpions, obtained his PhD, the results of which clearly changed the classification of the group. Unfortunately, Stockwell retired from scorpion studies shortly after getting his doctoral degree. Previous work on the phylogenetic classification of scorpions had already been performed by Lamoral [51] in South Africa, who also obtained his PhD in scorpion taxonomy. However, his phylogenetic analysis [52] was more modest than the one proposed by Stockwell.

Among European students, the preparation of a doctoral thesis using scorpions as models was rare until the 1970s. There are examples such as the thesis prepared by Couzijn [53] in the Netherlands on the taxonomy of the genus *Heterometrus* Ehrenberg of the family Scorpionidae, or that of Goyffon [54] in France using new biochemical approaches in taxonomy. Koch [55] in Australia can also be cited. Students from other regions equally appeared during this period, namely, L. F. Armas in Cuba, who still works with Caribbean scorpions, B. K. Tikader and D. B. Bastawade in India, R. Farzanpay in Iran or J. Santiago-Blay in the United States.

I am myself part of this generation. My first contact with M. Vachon was in 1971, and in 1972 he invited me to join the Laboratory of Zoology Arthropods in the Museum in Paris to start the preparation of my PhD dissertation under his direction. I accepted his offer, but had much field work to do before being able to synthesize a dissertation. I concluded my PhD dissertation in 1978 [56] and some years later obtained my Doctor’s Science degree [57]. By this time, Vachon had already been retired for several years and his assistance was less effective. Vachon died in 1991, three years after his last scientific publication [58].

### 1991 – 2014: the explosive number of scholars working with scorpions

From the 1990s to the present day, a remarkable ‘explosion’ in the number of scholars working on scorpions has been observed. Some of these experts, who had already been active since the 1980s, reasserted themselves during the following decades. Examples are L. Acosta in Argentina, A. Gromov in Russia, in addition to G. Lowe and V. Fet in the United States. Originally in Russia during the 1980s, V. Fet moved to the United States in 1988. However, a new generation of scorpion experts had emerged by the end of the 1990s. L. Prendini [59], originally from South Africa, moved to the United States, and led a very active group of students in New York City at the American Museum of Natural History. Among these can be cited E. Volschenk, V. Vignoli, A. Peretti, C. Mattoni and J. Ochoa etc.

In the year 2000 and beyond, an even greater number of young scholars dedicated themselves to scorpion studies. For example, B. E. Hendrixson, L. Exposito and M. R. Graham in the USA; L. Monod, B. Striffler, I. Stathi, B. Gantenbein in Europe; A. Ojanguren-Affilastro in Argentina; A. Giupponi and R. Pinto da Rocha in Brazil; R. Teruel in Cuba; C. Viquez in Costa Rica etc. One should not neglect either the Indian or especially the Chinese production. In China, under the direction of Prof. M.-S. Zhu, and partially with my collaboration, a number of students started to get training in scorpion research for the first time in that country’s history. Unfortunately, Prof. Zhu died in 2010, but several of his young Chinese scholars persist in their scorpion studies. In Mexico, O. F. Francke started the training of several new students. Many other young scholars are still receiving training in many parts of the world such as P. Sousa in Portugal, E. Ythier in France, A. Rossi in Italy, S. Loria and M. Webber in the USA. This list is not, however, exhaustive. Other less cited scholars also started their activities in this period (E. A. Yağmur in Turkey, O. Touloun in Morocco, R. Botero-Trujillo in Colombia, J.-M. Rojas-Runjaic in Venezuela etc.).

One question, however, can be asked: Are too many students receiving training to work on scorpion taxonomy and biology? The answer to this question could be yes, since many if not most of these young scholars leave the field of scorpiology after just a few years of research to dedicate themselves to other fields of biology. However, the basic training received during their doctoral formation is directed not only to scorpion studies but to a much larger array of possibilities. It is important to remember that doctoral training is not intended to train ‘scorpion experts’, but rather to train people for broader fields such as evolutionary biology, population biology, phylogeny and biogeography, etc. Scorpions are useful tools to address broader questions in many scientific fields. Today, a number of professional scholars are working in the United States, Europe, South America, and to a lesser extent, Asia and North Africa. Most certainly, the new generation of researchers will inevitably replace the older experts and can continue scorpion research after they retire.

### The importance of academic training

Academic training, such as achieving a doctoral degree, is fundamental in any field of biology. When I have discussions with students attracted to scorpions or other zoological groups, my first point is to explain that one does not prepare a doctoral dissertation on scorpions, spiders or any other zoological group. Instead, the aim of any doctoral dissertation is to train the student in one basic subject of biology, which may be evolutionary or population biology, phylogeny, biogeography, eco-physiology, genetics etc. Receiving the necessary biological basis/background is a critical aspect to any student who will be faced with many biological problems during his future years of research.

Because scorpions represent an ‘attractive zoological group’ (as also are spiders, birds, butterflies etc.) they draw the interest of many non-professional people who have fun observing and studying these animals. Since the 1980s and especially after the 1990s it is quite common to observe not only in European countries but also in North America numerous ‘exotic animal markets’. The real explosion of this (not always legal) business allowed many amateurs to have access to scorpions and other exotic animals. Many amateurs also make field trips and collect animals themselves; however, such expeditions are frequently carried out in the absence of any program of cooperation with the scholars of the visited countries. Several non-professionals are correct in their procedures and can even bring some useful contributions to the scientific community, by relaying useful information via websites or other media. However, the direct interference of non-academic persons in scientific results may provoke serious long-term problems. They produce results treating scorpions in a pure typological (or philatelic) approach. In these cases scorpions represent toys rather than tools in their research. The origin of the studied material is not always clear, and there are no permits proving that it was legally collected in their original countries. Many if not most emerging countries in which biodiversity is very important have severe laws concerning the collection and exportation of biological material. Field work carried out by academics normally abides by these laws, but non-academic people often ignore such rules.

As strongly recommended by the International Code of Zoological Nomenclature, type material, which represents the reference for all new taxa, should be exclusively deposited in official academic institutions. Many amateurs, however, disregard this rule and constitute important personal collections. Peer-reviewed journals are exhorted to reject publications when type material is not clearly deposited in an official institution, but again amateurs will exclusively publish their results in peripheral journals that generally neglect this rule. Faced with this situation, the best reaction from the academic community should be to neglect or at most pay little attention to this marginal pollution caused by non-academic people. As stated by Vanzolini [60] these contributions are worse than null because in many cases they are negative.

### The cooperation among scholars

Cooperation among people studying scorpions is not recent. At least as far back as the golden age, many contacts and exchanges took place between a certain numbers of experts such as E. Simon, R. I. Pocock, K. Kraepelin and T. Thorell. These contacts can be attested by the voluminous correspondence which took place between E. Simon and his European colleagues, and are still deposited in the Laboratory of Zoology Arthropods of the Museum in Paris. In contrast with what happens today, most of the correspondence in that era was conducted in French, which was then an international language. Travelling and visits made by these experts to other museums were, however, less frequent in those times, but some exceptions are known such as Kraepelin’s visit to the Museum in Paris in 1900. In subsequent years, travelling became more common and even some experts from overseas came to Europe, such as C. Mello-Leitão from the Brazilian National Museum in Rio de Janeiro who in the 1930s visited museums in several cities including Paris. After World War II and in the 1960s, M. Vachon, who had become a reference to young experts, started to receive many visits, from colleagues from different countries, in particular H. Stahnke, G. Levy, E. Maury, F. Matthiesen, B. Lamoral etc.

**Figure 2:**
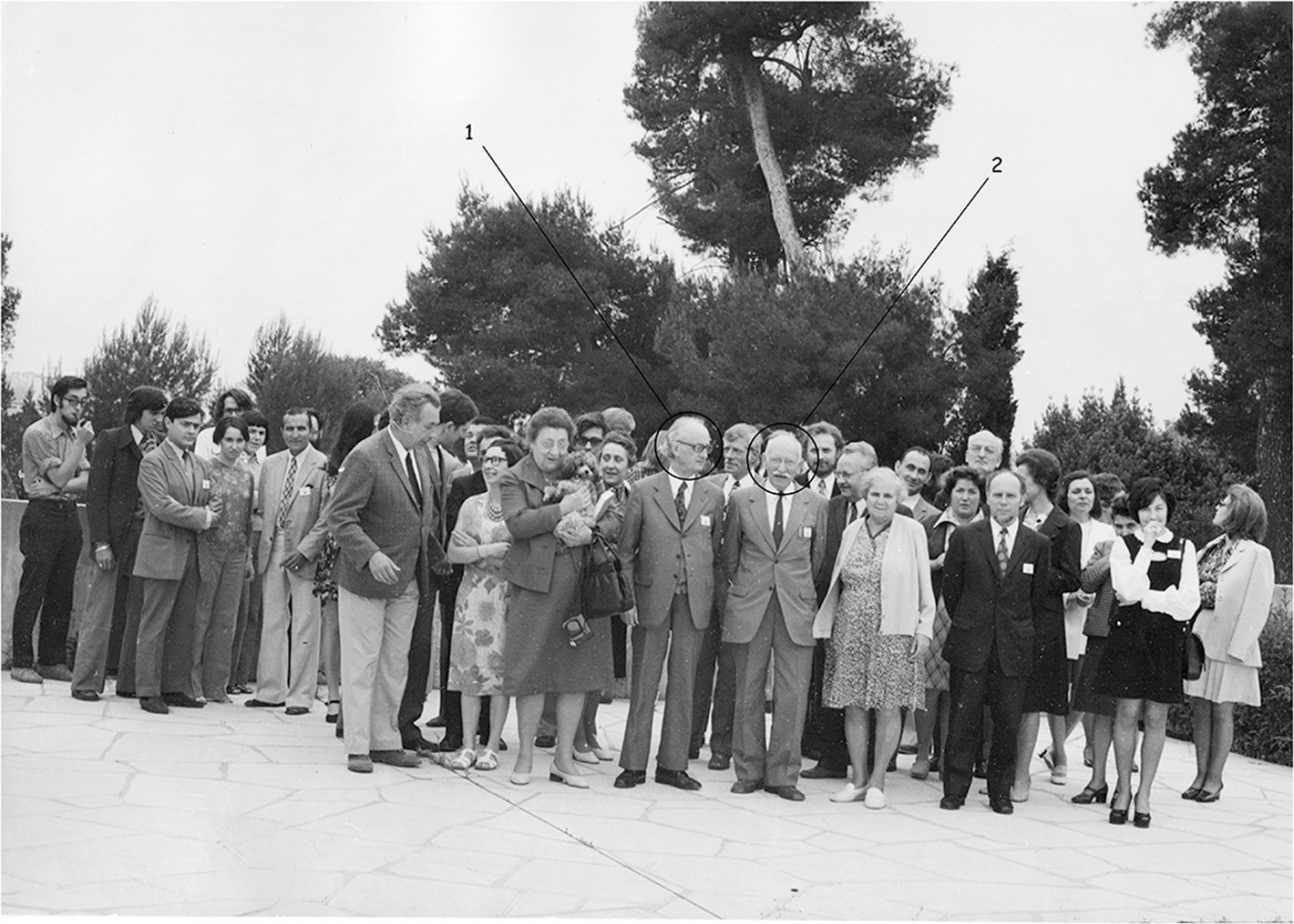
**One of the photos taken during the IV International Congress of Arachnology which took place in Paris in 1968.** M. Vachon (1) is next to P. Bonnet (2).

**Figure 3:**
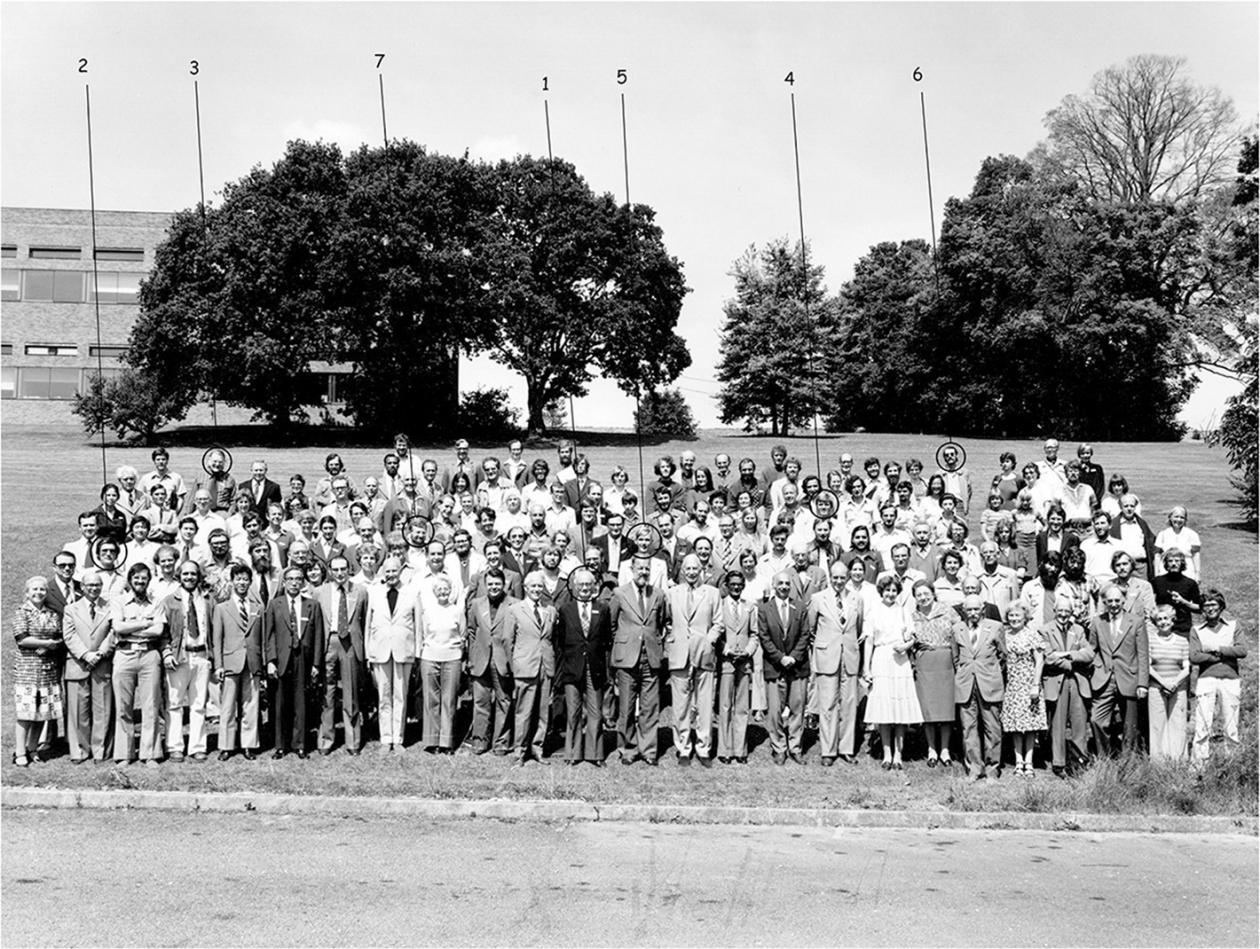
**Official photo of the VII International Congress of Arachnology which took place in Exeter (UK) in 1977.** A number of scorpiologists were present: (1) M. Vachon, (2) B. Lamoral, (3) J. L. Cloudsley-Thompson, (4) N. F. Hadley, (5) H. W. C. Couzijn, (6) W. R. Lourenço, (7) M. R. Warburg.

With the creation of the International Center of Arachnological Documentation (CIDA – Centre International de Documentation Arachnologique) by M. Vachon and O. Kraus (from Germany) in the early 1960s (today International Society of Arachnology) and with the promotion of international arachnological meetings, contact between scorpion experts became a reality. The first international congresses on arachnology, attended by several scorpion experts, took place in Paris in 1968 and in Exeter (UK) in 1977 (Figures 2 and 3). It was, however, under the influence of G. A. Polis that the first Symposium on Scorpions was organized and took place in 1985, as part of the National Meeting of the American Arachnological Society that was held in Los Angeles. This symposium was very successful, and assembled 12 experts from the United States, Europe and Israel. A second similar symposium was again organized by Polis and took place in 1991, as part of the American Society of Zoologists meeting which took place in Atlanta. Subsequently, other symposia dedicated to scorpions were organized but had a minor impact on scorpion research. During the first two meetings organized by Polis, some very positive ideas were considered and resulted in some important publications such as *The Biology of Scorpions*[47], *Scorpions Biology and Research *[61] and also the *Catalog of the Scorpions of the World*[62].

At the present, cooperation still exists, but in a much less spontaneous form than before. Groups studying scorpions form clans that are generally not open to other groups. This type of attitude can also generate conflicts among groups, which is not necessarily productive for research in general. One can only expect that this situation will evolve positively with time.

### Connections between zoologists and experts on venoms and toxins

If scorpions are fascinating animals, their appeal was from the very beginning connected with their notoriety as a ‘man killer’. Nonetheless, only a limited number of species, probably no more than 50 are actually responsible for serious or lethal incidents. It is true, however, that scorpions are responsible for a significant number of human deaths every year which is only surpassed by those caused by snakes and bees [47,61]. Most deadly species belong to the family Buthidae, however, species belonging to at least two other families, Hemiscorpiidae and Scorpionidae, also contain species posing a threat to humans.

The origin of mammal-specific toxins appears as an important issue in scorpion evolution. Old World lineages of Buthidae with very potent neurotoxic venom, such as the genera *Androctonus* Ehrenberg and *Leiurus* Ehrenberg, share separate mammal- and insect specialized neurotoxins that are specific for Na + channels [61]. Conversely, New World genera such as *Centruroides* Marx and *Tityus* C. L. Koch have potent toxins that act on both mammals and insects.

For some years now contacts have been established between zoologists and people whose research embraces scorpions in order to provide more general information about this zoological group. The idea is to demonstrate that the group’s diversity and distribution patterns are much more complex than it seems at first sight. If the group’s diversity is important, the same could be said about its diversity of toxins. Today only a very limited number of distinct taxa retain the attention of toxin experts, but perhaps this overview about scorpions may encourage the interest of researchers on biochemistry and molecular biology of venom toxins to expand their research to a broader array of scorpion groups, in particular those that can be informative on the evolution of complex venoms.

**Figure 4:**
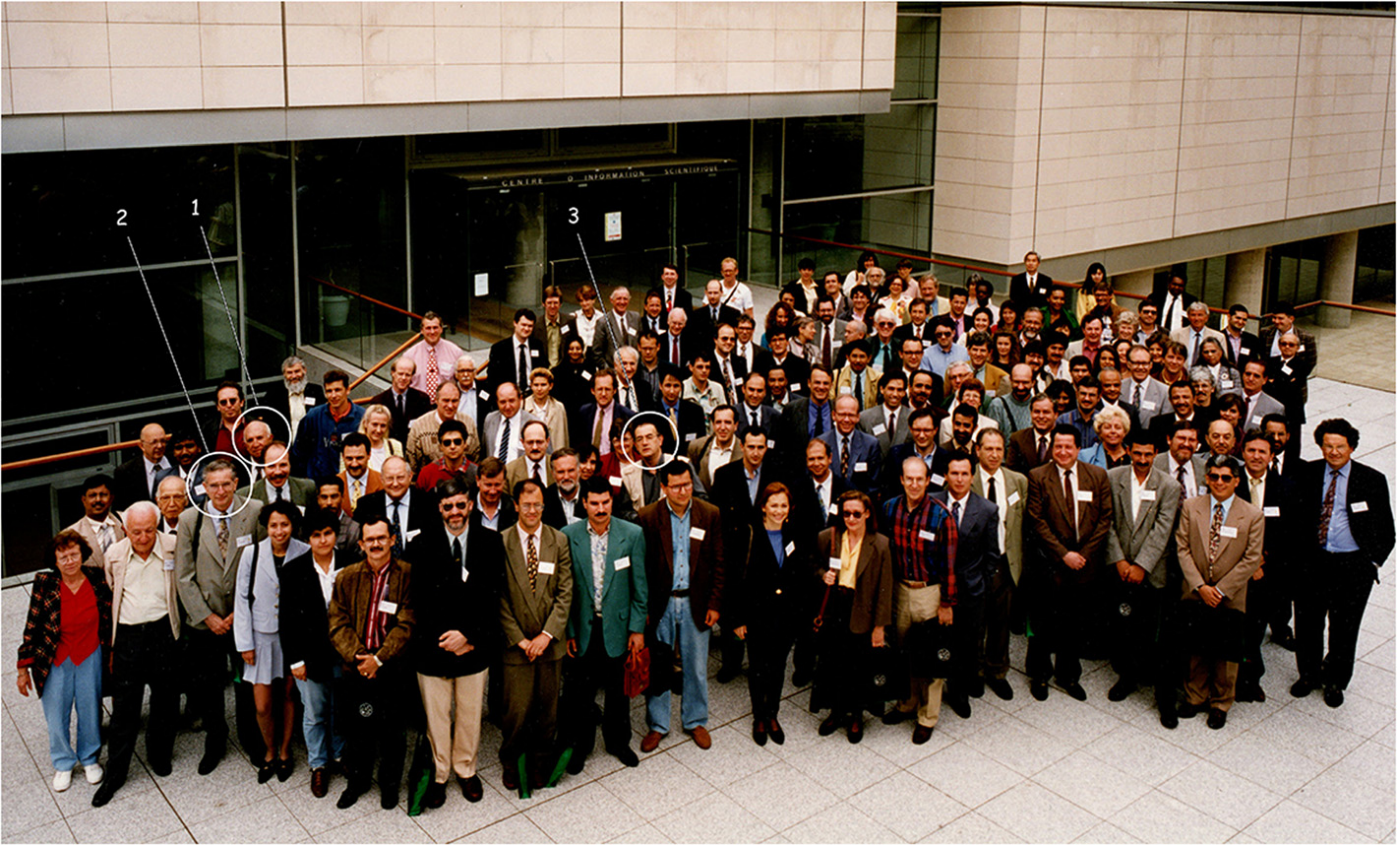
**Official photo of the 1**^**st **^**International Congress on Envenomations and Their Treatments which took place at the Pasteur Institute, Paris, in 1995.** (1) C. Diniz, (2) M. Goyffon, (3) W. R. Lourenço.

I personally developed collaborative exchanges with people working on venoms and toxins during the last 20 years. In the early 1970s while I was a PhD student I had the privilege to meet Prof. Carlos Diniz from the University of São Paulo, at Ribeirão Preto, a leading name in the study of scorpion toxins. After this preliminary contact, our paths diverged for more than 20 years. In 1995, we met again during the 1^st^ International Congress on Envenomations which was held in the Pasteur Institute in Paris (Figure 4). From this starting point, I was invited to organize special classes and conferences in several meetings of the Brazilian Society of Toxinology and of the International Society on Toxinology. These exchanges can be considered modest, but experiences such as these may open the door to more substantial exchanges between zoologists and venoms/toxins experts in the future.

## Conclusions

The aim of this communication is to provide general information about the history of scorpion studies in relatively recent times. The targets are readers who possibly use these organisms (or relatives of these organisms) in their own research, but are not aware of the history of researchers who have studied these organisms since the end of the 19^th^ century. This note is not exhaustive, and for more complete information about several classic authors, readers may refer to two very interesting publications [7,63]. The text was prepared to ensure clarity, accuracy and unambiguous communication, in the hope that its information may be accessible to a broad audience.

## Competing interests

The author declares that there are no competing interests.

## Authors’ information

Wilson R. Lourenço is an Emeritus Research Fellow at the National Museum of Natural History in Paris. He obtained his PhD in Evolutionary Biology from the University Pierre et Marie Curie (Paris VI) in 1978 and, his DSc in Population Biology, from this same University in 1985. He has worked since 1971 in the fields of taxonomy, biology, biogeography and ecology of scorpions. He has published over 600 papers, and several monographs and books. A field biologist, he collaborates with South American, African and Asian teams.

## References

[B1] Kraepelin K Dahl F Scorpiones und Pedipalpi Das Tierreich. Herausgegeben von der Deutschen zoologischen Gesellschaft 1899 Berlin: Verlag von R. Friedländer und Sohn 1 265

[B2] Calmette A Les venins: les animaux venimeux et la sérothérapie antivenimeuse 1907 London: Forgotten Books 411

[B3] Maurano HR O escorpionismo Doctor’s Degree Thesis 1915 Rio de Janeiro: Faculdade de Medicina do Rio de Janeiro

[B4] Koch CL Übersicht des Arachnidensystems 1 Band 1837 Nürnberg: CH Zeh’sche Buchhandlung 1 39

[B5] Peters MB Ueber eine neue Eintheilung der Skorpione und ueber die von ihm Mossambique Gessalmmelten Arten von Skorpionen Monatsh Dtsch Akad Wiss Berlin 1861 261 507 517

[B6] Lourenço WR Schwartz EF, de la Vega RC R, Possani LD Scorpion diversity and distribution; past and present patterns Handbook of Toxinology Volume Scorpions 2014 London: Springer Verlag (in press)

[B7] Bonnet P Bibliographia Araneorum: analyse méthodique de toute la literature aranéologique jusqu’en 1939 1945 Tome I. Toulose: Les Frères Douladoure 832

[B8] Thorell T On the classification of scorpions Ann Mag Nat Hist 1876 17 4 1 15

[B9] Simon E Simon E Scorpiones Les Arachnides de France. Tome septième, les ordres des chernetes, scorpiones et opiliones 1879 Paris: Librairie encyclopédique de Roret 332

[B10] Simon E Descriptions des genres et espèces de l’ordre des Scorpions Ann Soc Entomol Fr 1880 10 5 377 398

[B11] Kraepelin K Die Geographische verbreitung der scorpione Zool Jahr Abt Syst 1905 22 321 364

[B12] Pocock RI Notes on the classification of scorpions, followed by some observations upon synonymy, with descriptions of new genera and species Ann Mag Nat Hist 1893 12 6 303 330

[B13] Pocock RI Scorpions and their geographical distribution Nat Sci 1894 4 27 353 364

[B14] Banks N The scorpions of California Pomona College J Entomol 1910 2 2 185 190

[B15] Pavesi P de Carlini A A list of scorpions determined by P. Pavesi Rincoti ed aracnidi dell’isola di Cephalonia 1901 *Bull Soc Entomol Ital* 1901, **33**:75–79

[B16] Penther A Beitrag zur kenntnis amerikanischer skorpione Ann Naturhist Mus Wien 1913 27 239 252

[B17] Laurie M Notes on the anatomy of some scorpions and its bearing on the classification of the order Ann Mag Nat Hist 1896 17 6 185 193

[B18] Laurie M Further notes on the anatomy and development of Scorpions, and their bearing on the classification of the order Ann Mag Nat Hist 1896 18 6 121 133

[B19] Bialynitskii-Birula AA Arthogastric Arachnids of Caucasia Scorpiones. Part 1 1964 Jerusalem: Israel Program for Scientific Translations 170 Trad of: *Mém Mus Caucase*, Sér. A 1917a, **5**:1–253

[B20] Bialynitskii-Birula AA Fauna of Russia and Adjacent countries. Arachnoidea. Scorpiones 1965 Volume 1 Jerusalem: Israel Program for Scientific Translations 154 Trad of: Mus Zool Akad Sci Russie 1917b, 1–227

[B21] Borelli A Di alcuni scorpioni del Chile Rev Chil Hist Nat 1900 4 5 61 66

[B22] Borelli A Scorpioni raccolti dal dott. Filippo Silvestri nella Repubblica Argentina e regioni vicine Boll Mus Zool Anatom Comp Torino 1901 16 403 1 12

[B23] Pavlovsky EN On the morphology of the male genital apparatus in scorpions Trudy Leningradskogo Obshchestva Yestestvoispytatelei 1924 53 2 17 86 Trans. Leningrad Soc. Nat

[B24] Pavlovsky EN Zur morphologie des weiblichen genitalapparats und zur embryologie des Skorpione Ann Mus Zool I’Acad Sci I’URSS 1925 26 137 205

[B25] Baerg WJ Some poisonous arthropods of North and Central America4th Int Cong Ent Ithaca 1929 2 418 438

[B26] Banks N Scorpions and Pedipalpi collected by Dr. E. Mjoberg in Borneo Sarawak Mus J 1928 3 4 505 506

[B27] Chamberlin RV The northern range of the scorpion Science 1924 59 64 17806240 10.1126/science.59.1516.64-a

[B28] Hoffmann CC Monografias para la entomología médica de México. Monografia n° 2, Los escorpiones de México. Primera parte: Diplocentridae, Chactidae, Vaejovidae An Inst Biol Univ Nac Autón Méx 1931 2 4 291 408

[B29] Hoffmann CC Monografias para la entomología médica de México. Monografia N° 2, Los escorpiones de México. Segunda parte: Buthidae An Inst Biol Univ Nac Autón Méx 1932 3 3 243 282 (4):283–361

[B30] Hewitt J Facts and theories of the distribution of scorpions in South Africa Trans R Soc S Afr 1925 12 249 276

[B31] Glauert L New Victorian scorpion Victorian Nat 1930 47 7 109

[B32] Takashima H Scorpionida and Pedipalpi of the Japanese Empire Acta Arachnol 1943 8 5 30 [in Japanese]

[B33] Mello-Campos O Os escorpiões brasileiros Mem Inst Oswaldo Cruz 1924 17 2 237 363 10.1590/S0074-02761924000200002

[B34] Mello-Leitão C Revisão do gênero *Tityus* Physis (B Aires) 1939 17 57 76

[B35] Lutz A Mello EC Descrição de 5 espécies brasileiras dos gêneros *Tityus* e *Rhopalurus* Folha Méd 1922 3 4 25 26

[B36] Lourenço WR Sur les pas de Jean A. Vellard. A propos de sa contribution à l’étude des scorpions (*Chelicerata*) Rev Ib Arachnol 2001 3 25 36

[B37] Finnegan S Report on the scorpions collected by Mr. Bertram Thomas in Arabia J Linn Soc Zool London 1932 38 258 91 98 10.1111/j.1096-3642.1932.tb00695.x

[B38] Lourenço WR A new species of *Apistobuthus* Finnegan, 1932 *Chelicerata*, scorpiones, buthidae from Iran Entomol Mitt Zool Mus Hamburg 1998 12 157 237 244

[B39] Mello-Leitão C Escorpiões sul-americanos Arq Mus Nac Rio de Janeiro 1945 40 1 468

[B40] Vachon M Recherches anatomiques et biologiques sur la reproduction et le développement des pseudoscorpions Doctor’s Science Degree Thesis 1938 Paris: Faculté des sciences

[B41] Vachon M Voyage en A. O. F. de L. Berland et J. Millot V Bull Soc Zool France 1940 65 170 184

[B42] Vachon M Sur la systématique des scorpions Mém Mus Natl Hist Nat 1940 13 2 241 260

[B43] Vachon M Etudes sur les Scorpions 1952 Alge: Institut Pasteur d’Algérie 482 18877227

[B44] Matthiesen FA Parthenogenesis in scorpions Evolution 1962 16 2 255 256 10.2307/2406202

[B45] Levy G Amitai P Fauna Palaestina. Arachnida I: Scorpiones 1980 Jerusalem: Israel Academy of Sciences and Humanities 130

[B46] Francke OF Systematic revision of diplocentrid scorpions (Diplocentridae) from Circum-Caribbean lands 1978 Lubbock: Texas Tech University press (Series special publication, volume 14)

[B47] Polis GA Editor. The biology of scorpions 1990 Stanford: Stanford University press 587

[B48] Brownell P Polis GA Editors. Scorpion Biology and Research 2001 New York: Oxford University press 431

[B49] Sissom WD Systematics of the nitidulus group of the genus Vaejovis, with comments on phylogenetic relationships within the family Vaejovidae (Arachnida: Scorpiones) PhD Thesis 1985 Nashville: Vanderbilt University 292

[B50] Stockwell SA Revision of the phylogeny and higher classification of scorpions (Chelicerata) PhD Thesis 1989 Berkeley: University of California 319

[B51] Lamoral BH The scorpions of Namibia (Arachnida: Scorpionida) Ann Natal Mus 1979 23 3 497 784

[B52] Lamoral BH Gruber J A reappraisal of suprageneric classification of recent scorpions and of their zoogeography Internationaler Arachnologen-kongress abdgehalten ander Universitat fur Bodenkultur wien 1980 Volume 8 Viena: H. Egermann 439 444

[B53] Couzijn HWC Revision of the genus *Heterometrus* Hemprich & Ehrenberg (Scorpionidae, arachnidea) Zool Verh Rijk V Nat Hist Laiden 1981 184 1 196

[B54] Goyffon M Effets physiopathologiques de l’irradiation par radiations ionisantes chez le scorpion Doctor’s Science Degree Thesis 1975 Paris: Université Pierre et Marie Curie 189

[B55] Koch LE The taxonomy, geographic distribution and evolutionary radiation of Australo-Papuan scorpions Rec W Aust Mus 1977 5 2 83 367

[B56] Lourenço WR Etude sur les scorpions appartenant au ‘complexe’ Tityus trivittatus Kraepelin, 1898 et, en particulier de la sous-espèce Tityus trivittatus fasciolatus, Pessôa 1935 (Buthidae). Morphologie, systématique, répartition géographique, écologie, biologie générale et biologie sexuelle PhD Thesis 1978 Paris: Université Pierre et Marie Curie **1**:128

[B57] Lourenço WR Essai d’interprétation de la distribution du genre Opisthacanthus (Arachnida, Scorpiones, Ischnuridae) dans les régions néotropicale et afrotropicale. étude taxinomique, biogéographique, évolutive et écologique Doctor’s Science Degree Thesis 1985 Paris: Université Pierre et Marie Curie 287

[B58] Vachon M Abe T Colonization of the Krakatau Islands (Indonesia) by scorpions Acta Arachnol 1988 37 1 23 32 10.2476/asjaa.37.23

[B59] Prendini L Wheeler WC Scorpion higher phylogeny and classification, taxonomic anarchy, and standards for peer review in online publishing Cladistics 2005 21 5 446 494 10.1111/j.1096-0031.2005.00073.x 34892945

[B60] Vanzolini PE Zoologia sistemática, geografia e a origem das espécies 1970 São Paulo: Instituto de Geografia, USP 56 Série Teses e monografias, 3

[B61] Loret E Hammock B Brownell P, Polis GA Structure and neurotoxicity of venoms Scorpion Biology and Research 2001 New York: Oxford University Press 204 233

[B62] Fet V Sissom WD Lowe G Braunwalder ME Editors. Catalog of the Scorpions of the World (1758–1998) 2000 New York: New York Entomological Society 690

[B63] Savory TH Spiders, men, and scorpions. Being the history of arachnology 1961 London: University of London Press 191

